# Low-Density Lipoprotein Cholesterol Levels in Coronary Artery Disease Patients: Opportunities for Improvement

**DOI:** 10.1155/2022/7537510

**Published:** 2022-04-27

**Authors:** Gary J. Martin, Meron Teklu, Edwin Mandieka, Joe Feinglass

**Affiliations:** ^1^Department of Medicine, Northwestern University Feinberg School of Medicine, Chicago, IL, USA; ^2^Northwestern University Feinberg School of Medicine, Chicago, IL, USA; ^3^Internal Medicine Resident, McGaw Medical Center of Northwestern University, Department of Medicine, Chicago, IL, USA; ^4^Division of General Internal Medicine and Geriatrics, Northwestern University Feinberg School of Medicine, Chicago, IL, USA

## Abstract

**Background:**

We sought to characterize the level of LDL-C control and identify opportunities for improvement and characteristics of patients who were undertreated.

**Methods:**

Study patients were from a large multihospital system, age <90, with documentation of at least two encounters with a CAD diagnosis or procedure before a first measured LDL-C level and a last recorded LDL-C measurement over a minimum six-month (median = 22 months, IQR = 15–26 months) follow-up from January 2017 to September 2019. Linear regression analysis for last recorded LDL-C level was used to analyze the effects of statin intensity and patient characteristics.

**Results:**

Among 15,111 eligible patients, mean age was 68.4 (SD = 10.8), 68.7% were male, and 79.4% were non-Hispanic White. At follow-up, 87.8% of patients were prescribed a statin, 9.7% were on ezetimibe, and 0.5% were on a PCSK9 inhibitor. Mean LDL-C at follow-up was 75.6 mg/dL and 45.5% of patients were on high-intensity treatment. Higher LDL-C values were associated with female sex, younger patients, non-Hispanic Black patients, high poverty or out of state zip code, Medicaid, or angina as the qualifying diagnosis. For 332 clinicians with >10 patients in the cohort, mean last recorded LDL-C values ranged from 47 to 102 mg/dL.

**Conclusions:**

There were important variations in LDL-C control between patients in our health system with the same indication for treatment. Variation in treatment among physicians is an area ripe for quality improvement interventions. This study may be easily reproduced by other medical centers and used for highlighting both patient and physician opportunities for improvement.

## 1. Introduction

LDL-C levels have long been linked to cardiovascular events [1]. Statins are well documented for secondary prevention dating back to the 4S trial [2]. Progressively lower LDL-C levels have continued to show incremental benefits [3] including the utility of nonstatin approaches [4]. Yet, many patients with established coronary artery disease (CAD) are undertreated leaving many opportunities for improvement [5–8]. Intensity of statin or other lipid-lowering medication use is known to be highly variable, but associated LDL-C outcomes have rarely been analyzed since newer treatments have been validated [6]. There are eight classes of cholesterol lowering drugs with various mechanisms of action [9, 10], but this study was focused on the three most widely used in current practice: statins, ezetimibe, and PCSK9 inhibitors.

The aim of this study was to identify patients in a large multihospital system with documented CAD who could be followed using electronic health records linked over time that allowed a reasonable opportunity to have their LDL-C controlled. We sought to characterize the level of LDL-C control, identify where there were opportunities for improvement, and what characterized patients who were undertreated. The study was designed to inform interventions to improve LDL-C levels in appropriate patients with the goal of reducing subsequent cardiovascular events and improving mortality.

## 2. Patients and Methods

Data were obtained from the health system's Enterprise Data Warehouse which combines electronic health records with patient administrative data for both inpatient and outpatient visits. Study inclusion required age <90, documentation of at least two encounters with a CAD diagnosis, or procedure code (ICD-10 diagnosis codes I20-I25, ICD-10 PCS procedure codes Z95.1 or Z98.61) before an index LDL-C level, as well as a subsequent follow-up LDL-C measured at least six months later. Cohort inception was based on an index LDL-C measurement after January 1, 2017, allowing for a two-year look back to 2015 to ascertain at least two encounters with a CAD diagnosis before the inception date. Cohort accrual continued from June 2018 to September 2019, allowing up to a 33-month follow-up period to obtain a last recorded LDL-C measurement. The last measured LDL-C during the follow-up period was used for analysis. Similarly, the last recorded prescription for lipid-lowering medication before the follow-up LDL-C measurement was analyzed. This study was approved by the IRB of our institution (STU00210370).

Patient demographic data included age (<60, 60–69, 70–79, 80, or older), sex and race, and ethnicity, defined as non-Hispanic White, non-Hispanic Black, Hispanic, or other/unknown. Insurance status, ascertained at cohort inception, included Medicare, Medicaid, private, or other/unknown. Patient zip codes were matched to American Community Survey 2019 five-year file census zip code tabulation areas (ZCTAs) estimates of the proportion of families living at or below poverty level. ZCTA poverty levels were characterized as <3%, 3–7.99%, 8% or more, or out of state. CAD diagnoses were hierarchically defined as coronary bypass or angioplasty procedure, myocardial infarction (MI), other CAD diagnoses, or angina only. Patients' body mass index (BMI) was obtained at cohort inception. Statin intensity at each patients' last recorded prescription was analyzed based on American Heart Association guidelines [11] and categorized as high, moderate, low, unclassified (when data did not permit clear assignment), or no lipid-lowering medication prescribed. In the case of ezetimibe added to a statin, we assumed an additional 20% lowering of the LDL-C to arrive at the intensity category.

Chi square tests were used to determine the statistical significance of associations between patient demographics and clinical characteristics and last recorded LDL-C level (classified as <70, 70–99, 100–129, and 130 or more to convert mg/dL to SI in mmol/L multiplied by 0.02586). A multiple linear regression was estimated for (normally distributed) follow-up LDL-C, controlling for patient clinical and sociodemographic characteristics and with standard errors adjusted for clustering of patients within the last recorded LDL-C ordering physicians. Analyses were conducted with Stata Version 15 (College Station, TX) and IBM SPSS Version 27 (Armonk, NY).

## 3. Results

**Figure 1:**
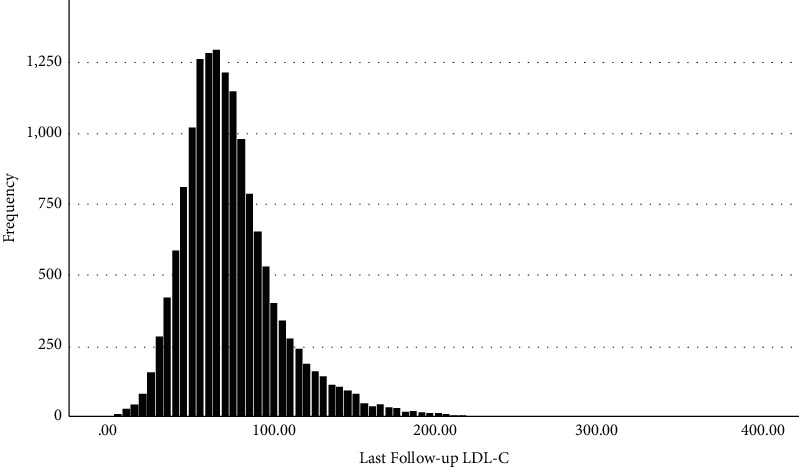
Distribution of final follow-up low-density lipoprotein cholesterol mg/dL levels for 15,111 health system patients with documented coronary artery disease.

There were 15,111 patients identified who had an index and last recorded LDL-C level measured during a mean follow-up time of 20.8 (SD = 6.9) months.  Figure 1 illustrates the distribution of last recorded follow-up LDL-C values. The mean LDL-C at last recorded follow-up was 75.6 (SD = 30.6) mg/dL. Males were 68.7% of the sample with an average LDL-C at follow up of 71.8 mg/dL vs. 83.8 mg/dL for females (Table 1). Higher LDL-C values were associated with younger age, non-Hispanic Black, high poverty or unknown zip code, Medicaid, or angina as the qualifying diagnosis. Looking at those on high (45.5% of the cohort), moderate, and low intensity treatment, follow-up LDL-C values were 70.4, 73.2, and 82.6, respectively. Overall, 87.8% of patients were on a statin, 9.7% were on ezetimibe, and 0.5% prescribed a PCSK9 inhibitor. A total of 11.5% of all sample patients had no electronic health record recorded lipid-lowering drug therapy prescription between their index and follow-up LDL-C measurement.

There was a subgroup of 881 patients with follow-up levels of >130 mg/dL. These patients were more likely to be female, non-Hispanic Black, and younger. Of those with a follow-up LDL-C >130 mg/dL, 40.4% had no recorded lipid-lowering drug therapy prescription in their medical record.  Table 2 presents results of the linear regression analysis of follow-up LDL-C. Males had an expected LDL-C level 9.5 mg/dL lower than females, older patients had LDL-C levels significantly lower than expected compared to patients aged 59 or younger. Non-Hispanic Black identity was associated with a 6.2 mg/dL higher LDL-C as compared to non-Hispanic White. Also, compared to other CAD diagnoses, an MI diagnosis was associated with −4.3 mg/dL lower LDL-C, having a prior revascularization procedure with −3.3 mg/dL lower LDL-C.

**Figure 2:**
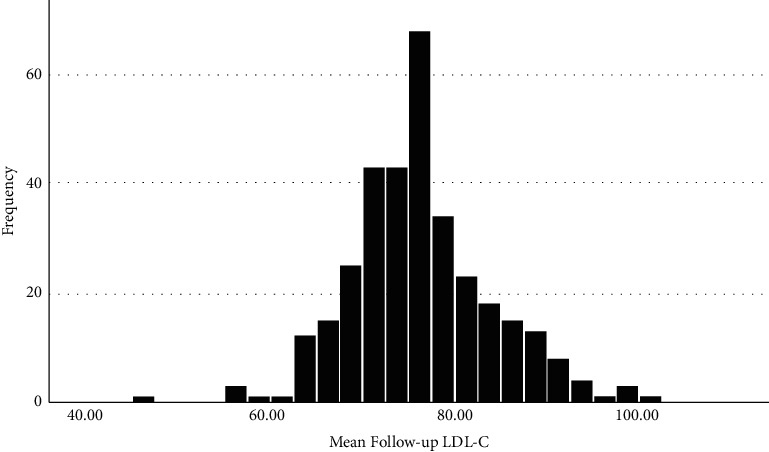
Distribution of mean final follow-up low-density lipoprotein cholesterol mg/dL among 332 health system physicians with at least 10 study patients (*N* = 12,888 study patients).

Treatment intensity was strongly associated with follow-up LDL-C level. As compared to those with high-intensity treatment, having no documented lipid-lowering prescriptions was associated with a 33.1 mg/dL higher LDL-C. Differences between moderate and low intensity patients were more modest but highly significant (*p* < 0.001).  Figure 2 reflects the mean last recorded LDL-C distribution for patients grouped by the managing physician for the 332 ordering physicians with at least 10 patients in the study cohort. While representing 26% of the total of 1,277 last recorded LDL-C ordering physicians, these 332 physicians cared for 12,899 patients or 85.3% of sample patients.

### 3.1. Limitations

This study of a selected health system population was focused on LDL-C and statin dose information and cannot assess patients' medication adherence. Although national data suggest adherence to statins has improved over time, sex and racial disparities persist in both screening and treatment [12, 13]. Side effects from a prior higher dose of statin could have also limited clinicians from prescribing the recommended intensity, but there is evidence that only represents about 5% of patients [13]. Our focus was on LDL-C levels but clinical impact could be further enhanced by measuring apolipoprotein B [14]. Finally, our electronic health record study is subject to documentation biases. We required multiple documented visits over time after a baseline LDL-C measurement and thus will be missing other patients with less complete ascertainment of CAD diagnoses or sequential LDL-C measurement.

## 4. Discussion

Utilizing our Enterprise Data Warehouse, we were able to identify a large diverse cohort of patients with CAD, their level of LDL-C control, intensity of treatment, and characteristics of those less likely to be well controlled. We also discovered a wide range of mean LDL-C levels between the patients cared for by various physicians. The value of secondary prevention in patients with CAD is well established. The 4S trial [2] was an early demonstration of the impact of LDL-C reduction where simvastatin reduced LDL-C from an average of 187 mg/dL down to 122 mg/dL. resulting in a reduction in mortality and major cardiac events (MCEs). More recent studies with lower LDL-C levels have reinforced this concept including newer therapies using PCSK9 inhibitors and ezetimibe. Our diverse sample shows some use of these newer therapies as data supporting their impact became available. Ezetimibe was shown to have an additive outcome benefit in 2015 [15]. Evolocumab data on outcomes for example were available in 2017 [16]. That study followed 27,564 patients with atherosclerotic cardiovascular disease (ASCVD) with an LDL-C > 70 mg/dL (averaging LDL-C 92 mg/dL on their baseline statin) being lowered to 30 mg/dL with the addition of the PCSK9i and an associated benefit in MCEs. Of course, there are many variables that affect individual responses and cardiovascular risks. Besides traditional risk factors, genetic predispositions range from endothelial dysfunction [17, 18] to variations in gastrointestinal absorption of lipids [19]. The goal of precision medicine in the future will be to include these individual characteristics in therapeutic choices.

Although high-intensity statin therapy has been widely recommended, the optimal LDL-C level has been controversial as has even the concept of a target, threshold, or goal LDL level [20]. There is increasing support for lower is better even down to less than 39 mg/dL [21, 22]. Serial intravascular ultrasound data [23], PCSK9i data [16, 24], and recent titration trials [25]support the incremental value of LDL-C values less than 60–70 mg/dL. In the Treat Stroke to Target trial [25], approximately one-third of study patients used ezetimibe in addition to the statin to reach that target. Higher intensity statin therapy is associated with better outcomes but appears to be underutilized [13]. Yao et al. noted improvement over time, but by 2016, it was still only used in 49.2% of ASCVD patients. Arnold et al. [5] found similar underutilization of high-intensity statins in the GOULD database. Overall statin use was 87%; 48.5% were on high-intensity statins, and 9.7% were on ezetimibe.

There are many barriers to improve LDL-C control. There is considerable variability in LDL-C reduction among patients on the same intensity of statin therapy [26]. Cost, despite the wide availability of generic high potency statins and ezetimibe, may be a limiting factor. Patients may fear side effects of higher doses. Various reasons for patients' nonadherence have been categorized by others [27].  Figure 2 demonstrates a range of average LDL-C for patients cared for by health system physicians. Physician inertia and workload may contribute as well as patient provider discordance. Lack of knowledge on the value of additional lowering of LDL-C levels may also limit optimal use. Some of the difference between physicians may represent the social determinates of health associated with the patients they care for in their particular office. If high-intensity statins (or maximally tolerated intensity) were not enough, ezetimibe may not have been considered as an option even though it was generically available. The expense and logistics of adding PCSK9 inhibitors are certainly limiting factors too but may be more justified in the very high-risk subgroup. Inclisiran is an option that could improve adherence but will be expensive and awaits clinically significant outcome data [28].

## 5. Conclusions

Many interventions have been studied to influence clinicians and improve quality [29]. Potential interventions include outreach to patients and providers, enlisting community leaders to advocate for healthy behaviors, audit and feedback report cards available to peers (or even publicly available), and EMR alerts. Our institution has had success with peer report cards and other feedback interventions [30, 31] and EMR alerts [32] for example. For LDL-C management, we plan on several of these approaches. LDL-C management is a shared responsibility. Arnold et al. noted in their recently established GOULD database that the care of postmyocardial infarction patients was managed just as often by primary care physicians as cardiologists [5]. Confusion to which clinician is in charge may be a factor as well [33].

Disparity in the control of cholesterol by sex and race has been found in other studies [7, 13, 34–36]. Several studies have found women to be disproportionately undertreated as we did [6, 7, 13, 34, 35]. Yao et al. also noted individuals identifying as Blacks or Hispanics were 10% less likely to be on a statin at 30 days after an index event [13]. Zhang et al. [37] have highlighted the challenges in racial disparities even after quality improvement efforts. The value of doing better for our patients is clear. For example, one can estimate the reduction in future CVD events; if we shifted CAD patients from an LDL-C of approximately 99 mg/dL down to 60 mg/dL, we could reduce risk an additional 17% [3]. The opportunities for improvement in secondary prevention are even greater in the peripheral vascular disease population [38].

There are important variations in LDL-C control between patients with the same indication for treatment. Variation among physicians in controlling LDL-C is also apparent and this analysis is unique. We feel this is another area ripe for analysis and intervention. Future research using qualitative assessments of under treatment is a logical next step. We plan on a spectrum of interventions at our institution including an EMR alert to clinicians, short educational outreach to clinicians, and feedback reports with specific performance information comparing the clinician with peers and the recommended threshold. We may consider outreach to patients as well in order to activate them in the process [39, 40]. This approach can be used by other health systems or insurers with electronic records for developing both the physician and patient facing interventions that improve quality of care and health equity.

## Figures and Tables

**Table 1:** Last measured low-density lipoprotein cholesterol mg/dl levels. *N* = 15,111 health system patients with documented coronary artery disease (CAD).

	Sample percent	Mean (SD) final LDL-C	Percent <70, *N* = 7302	Percent 70–99, *N* = 5314	Percent 100–129, *N* = 1614	Percent 130 plus *N* = 881
Female	30.3	83.8 (34.6)	38.0	36.8	15.3	9.8
Male	68.7	71.8 (27.7)	53.0	34.4	8.6	4.0

Age						
<60	20.3	80.7 (33.4)	40.9	36.8	13.9	8.4
60–69	31.6	76.5 (30.6)	46.8	36.3	10.8	6.1
70–79	32.5	72.8 (29.2)	52.1	34.5	8.9	4.5
80+	15.6	72.7 (28.5)	53.2	32.3	9.8	4.7

Race and ethnicity						
Non-Hispanic White	79.4	74.6 (29.5)	49.2	35.5	10.0	5.3
Non-Hispanic Black	8.3	84.5 (35.7)	37.2	36.7	15.8	10.3
Hispanic	4.8	76.8 (33.6)	46.7	33.6	12.2	7.5
Others/unknown	7.5	74.5 (31.6)	51.9	31.2	10.9	6.0

Zip code census area poverty level						
<3% of families	23.8	74.2 (28.6)	49.3	35.5	10.1	5.1
3–7.99% of families	50.7	75.0 (30.1)	49.2	35.3	10.2	5.4
8% or more of families	20.9	78.3 (33.5)	45.6	34.3	12.4	7.7
Out of state/unknown	4.6	78.2 (30.9)	45.9	36.5	11.1	6.4

Insurance status						
Private	27.4	78.3 (31.6)	44.2	36.9	11.7	7.2
Medicaid/uninsured	4.0	80.4 (36.4)	43.4	36.5	11.7	8.4
Medicare	68.6	74.2 (29.7)	50.3	34.5	10.1	5.2

Body mass index						
Underweight	0.5	75.0 (31.7)	52.1	27.4	16.4	4.1
Normal weight	13.2	77.2 (30.8)	46.2	35.5	12.2	6.1
Overweight	44.1	75.1 (30.0)	49.2	35.0	10.2	5.6
Obese	35.3	75.0 (30.1)	48.3	35.7	10.4	5.6
Morbid obese	6.2	78.7 (34.5)	46.4	33.5	12.0	8.1
BMI missing	0.7	74.6 (28.8)	49.0	32.4	9.8	8.8

CAD diagnosis						
Angina only	5.7	82.6 (33.6)	40.8	34.0	15.3	9.9
Other CAD diagnoses only	55.3	77.6 (30.8)	44.9	36.9	11.8	6.5
MI only	9.5	73.5 (31.1)	52.5	31.0	11.3	5.2
Bypass or angioplasty procedure	29.6	70.9 (28.6)	54.9	33.5	7.5	4.1

Statin intensity						
High	45.5	70.4 (27.9)	55.5	33.6	7.3	3.7
Moderate	36.9	73.3 (26.2)	49.5	37.7	9.4	3.4
Low	4.6	82.6 (31.2)	36.2	33.5	15.7	7.3
Unclassified	1.5	85.6 (38.6)	39.8	33.5	15.9	10.7
No prescription	11.5	99.1 (39.3)	22.4	31.3	25.6	20.6

All comparisons *p* < 0.01.

**Table 2:** Linear regression results for the last measured low-density lipoprotein cholesterol mg/dL level. *N* = 15,111 health system patients with documented coronary artery disease (CAD)^*∗*^.

	B	Se	*p* value
Female			
Male	−9.55	0.52	<0.0001

Age			
<60	Reference		
60–69	−3.25	0.77	<0.0001
70–79	−7.37	0.84	<0.0001
80+	−8.87	0.96	<0.0001

Race and ethnicity			
Non-Hispanic White	Reference		
Non-Hispanic Black	6.22	0.93	<0.0001
Hispanic	0.17	1.12	0.88
Others/unknown	−0.28	0.89	0.75

Zip code census area poverty level			
<3% of families	Reference		
3–7.99% of families	0.48	0.57	0.40
8% or more of families	1.15	0.74	0.12
Out of state/unknown	1.61	1.17	0.17

Insurance status			
Private	Reference		
Medicaid/uninsured	0.09	1.25	0.94
Medicare	−1.39	0.70	.06

Body mass index			
Underweight	−6.31	2.99	0.03
Normal weight	Reference		
Overweight	0.46	0.70	0.51
Obese	−0.40	0.76	0.56
Morbid obese	−0.42	1.20	0.73
BMI missing	−7.00	3.61	0.05

CAD diagnosis			
Angina only	1.28	1.10	0.24
Other CAD diagnoses only	Reference		
MI only	−4.2	0.88	<0.0001
Bypass or angioplasty procedure	−3.2	0.56	<0.0001

Statin intensity			
High	Reference		
Moderate	2.89	0.54	<0.0001
Low	9.23	1.49	<0.0001
Unclassified	13.31	1.53	<0.0001
No prescription	33.12	1.21	<0.0001

^*∗*^Standard errors adjusted for clustering of patients within final LDL-C ordering physician.

## Data Availability

The data that support the findings of this study are available from the corresponding author upon reasonable request.
